# Supplementary material for: Burnout Syndrome among Pediatric Nephrologists—Report on Its Prevalence, Severity, and Predisposing Factors
Source: Medicina (Kaunas). 2022 Mar 18;58(3):446. doi: 10.3390/medicina58030446 (PMC8950474; doi:10.3390/medicina58030446)
Supplement: Supplementary file 1 [file medicina-58-00446-s001.zip › Pawlowicz-Szlarska et al. Suppl. File 1. The study survey..pdf]

Dear Participant,

Burnout syndrome is considered as a serious problem among healthcare professionals. It was already confirmed in many studies that burnout may significantly affect patients' satisfaction, physicians' practice and clinical performance and may lead to resignations from work.

At the moment there are no reliable data on burnout syndrome among polish pediatric nephrologists – both among specialists and physicians in-training. Proper measuring and assessing burnout syndrome is a key to success in creating adequate remedy programs.

We would like to ask to take a few minutes and share your experiences in our survey study addressed to all physicians working within pediatric nephrology.
The survey is completely anonymous, and analyzed data will be used scientifically and for creating the burnout remedy program responding for actual needs.

By completing the survey, participants are stating consent to participate in the study.

This project is the result of cooperation between the Young Nephrologists' Club of the Polish Society of Nephrology and the Young Nephrologists' Forum of the Polish Society of Pediatric Nephrology.

Thank you in advance for taking part in the project.

Best regards,
Piotr Skrzypczyk MD, PhD
Young Nephrologists' Forum of the Polish Society of Pediatric Nephrology
Ewa Pawłowicz, MD
Young Nephrologists' Club of the Polish Society of Nephrology

Burnout syndrome assessment (abbreviated Maslach Burnout Inventory)

	Every day	A few times a week	Once a week	A few times a month	Once a month or less	A few times a year or less	Never
I deal very effectively with the problems of patients.							
I feel I treat some patients as if they were impersonal objects.							
I feel emotionally drained from my work.							
I feel fatigued when I get up in the morning and have to face another day on my job.							
I've become more callous toward people since I took my job.							
I feel I am positively influencing other people's lifes through my work.							
Working with people all day is really a strain for me.							
I don't really care what happens to some patients.							
I feel exhilarated after working closely with my patients.							

Do you feel burned out?

- ☐ yes
- ☐ rather yes
- ☐ rather no
- ☐ no

Do you participate now in the burnout remedy or/and prevention program?

☐ yes ☐ no

If no, would you like to participate in burnout remedy or/and prevention program?

☐ yes ☐ no

If so, such program should be:

- ☐ group meetings
- ☐ individual meetings

If so, such program should be:

- ☐ obligatory for those who were diagnosed with burnout syndrome, founded by the employer
- ☐ voluntary, but founded by the employer
- ☐ voluntary, financed by the own resources of the doctor

Do you apply any strategies to combat burnout on your own?

☐ no ☐ yes

7. If so, what do you do?

- ☐ physical activity
- ☐ meetings with family/friends
- ☐ listening to/playing music
- ☐ sleeping
- ☐ isolating myself from others
- ☐ going to cinema
- ☐ other, please specify..

What contributes most to your burnout? [indicate maximum 3 best fitting options]?

- ☐ too many bureaucratic tasks
- ☐ spending too much time at work
- ☐ lack of respect from administrators/employers/staff/colleagues
- ☐ lack of respect from patients
- ☐ work overload leading to rush
- ☐ increasing computerization of practice
- ☐ insufficient compensation
- ☐ other, please specify..

Work-related and demographic data

Gender:

☐ female ☐ male

Age:

☐ <30 years old
☐ 30-50 years old
☐ 51-65 years old
☐ > 65 years old

Years of professional experience:
…………………… years

I am:

☐ pediatric nephrologist (board-certified specialist)
☐ pediatrician (board-certified specialist)
☐ specialist in pediatrics in-training in pediatric nephrology
☐ in-training doctor/resident (in pediatrics)
☐ in-training doctor/resident (in pediatric nephrology)
☐ other, please specify…………………………………………………………....

I work in: [mark every setting that fits]

☐ hospital (pediatric nephrology ward)
☐ hospital (pediatric ward)
☐ out-patient clinic (pediatric nephrology)
☐ out-patient clinic (pediatric)
☐ dialysis unit
☐ emergency department

I work in: [mark only one place that is your main workplace e.g. you spend most hours]

☐ hospital (pediatric nephrology ward)
☐ hospital (pediatric ward)
☐ out-patient clinic (pediatric nephrology)
☐ out-patient clinic (pediatric)
☐ dialysis unit
☐ emergency department

How many hours per week do you spend at work?

☐ not more than 40 hours
☐ 41-50
☐ 51-60
☐ 61-75
☐ more than 75 hours

Did you take your holiday leave last year?

☐ yes, I used all days
☐ yes, partially
☐ not at all

Supplementary File 1. The study survey.

Article title: Burnout syndrome among pediatric nephrologists – report on its prevalence, severity, and predisposing factors
Journal name: Pediatric Nephrology
Author names: Ewa Pawłowicz-Szlarska, Piotr Skrzypczyk, Małgorzata Stańczyk, Małgorzata Pańczyk-Tomaszewska, Michał Nowicki
Corresponding author:
Prof. Michał Nowicki, MD, PhD
Department of Nephrology, Hypertension and Transplantation
Medical University of Lodz
e-mail: michal.nowicki@umed.lodz.pl
